# Everyday life during the childhood cancer trajectory—childhood cancer survivors' descriptions of the role of caring support

**DOI:** 10.3389/fresc.2023.1098933

**Published:** 2023-06-16

**Authors:** Margaretha Stenmarker, Maria Björk, Marie Golsäter, Karin Enskär

**Affiliations:** ^1^ Institute of Clinical Sciences, Sahlgrenska Academy, University of Gothenburg, Gothenburg, Sweden; ^2^ Department of Pediatrics, Region Jönköping County, Jönköping, Sweden; ^3^ Department of Biomedical and Clinical Sciences, Faculty of Medicine and Health Sciences, Linköping University, Linköping, Sweden; ^4^ School of Health and Welfare, Jönköping University, Jönköping, Sweden; ^5^ CHILD—Research Group, School of Health and Welfare, Jönköping University, Jönköping, Sweden; ^6^ Child Health Services, Region Jönköping County, Jönköping, Sweden; ^7^ Department of Women’s and Children’s Health, Uppsala University, Uppsala, Sweden

**Keywords:** childhood cancer survivor, child, adolescents, swanson caring theory, participation, every day life, long term health conditions

## Abstract

**Background:**

Being diagnosed with cancer in childhood often has a direct impact on the child's opportunities to participate in activities and the child's sense of belonging in different life situations. Experiences of illness in youth affect the lives of these individuals in numerous ways and they need pronounced support to regain their normal life after treatment.

**Purpose:**

To illustrate how childhood cancer survivors describe the role of the caring support provided by healthcare professionals at diagnosis and during the cancer trajectory.

**Methods:**

A mixed methods approach was applied. Swanson's Theory of Caring was used to deductively analyze the answers in a study-specific questionnaire with Likert scales (1–5). Descriptive and comparative statistics and exploratory factor analyses were performed.

**Results:**

Sixty-two former patients, diagnosed with solid tumors/lymphoma in 1983 to 2003 in Sweden, participated. The mean time passed since treatment was 15.7 years. Swanson's caring processes Being with and Doing for were the most prominent loading categorical factor indicators. Higher scores for healthcare professionals being emotionally present (Being with), doing for others what they would do for themselves (Doing for) and being willing to understand the sick child's situation (Knowing) were highlighted by survivors older than 30 years, compared to those younger than 30 years (*p* = 0.041, *p* = 0.045, and *p* = 0.013, respectively). An increased vulnerability regarding their ability to cope with difficulties (Maintain belief) was found among participants who were treated during adolescence, related to schoolchildren (*p* = 0.048), and among those who had been treated with extra-cranial irradiation in comparison with no extra-cranial irradiation (*p* = 0.004). The role of having a partner in comparison with being single was underlined among those who felt they had acquired the tools they needed to take care of themselves (Enabling) (*p* = 0.013). The total explained variance was 63%.

**Conclusions:**

A person-centered care approach during treatment for childhood cancer, reflected by a caring model, highlights the role of healthcare professionals being emotionally present, involving children, performing actions, and with an approach that has potential long-term implications. Childhood cancer patients and survivors need not only clinically competent professionals, but professionals who provide caring interactions with compassion.

## Introduction

Long-term survivors of childhood cancer experience physical problems, psychological issues, as well as activity and participation challenges following treatment completion (1, 2). There has been growing attention to these individuals and their experiences of illness in childhood, during growth and development, but also how these circumstances have affected the survivors’ lives in numerous ways (3).

Over 80% of children diagnosed with cancer are now cured (4, 5) and they will become long-term survivors with many potential years of life ahead of them (6). In Sweden, the number of childhood cancer survivors is approximately 11 000, and among these survivors the majority, about 70%, have developed late complications (7). Despite this, it is important to not just focus on their illness and late complications but also on their health. Health is a resource for everyday life and a positive concept emphasizing social and personal resources as well as physical capabilities (WHO, 1986) (8). Health is related to the child's ability to perform the activities he/she wants, but also to the possibility to participate in an everyday life (9). When children participate in different forms of activities, it positively influences their health, development and well-being (10, 11). Still, the physical and social environment as well as limited resources can act as barriers limiting participation (12). If healthcare personnel are to be able to give all of these affected individuals good person-centered care, they need to start with the specific person they have in front of them and to identify their various needs, resources and experiences (13). However, Sundler et al. (2020) highlights that a person-centered approach to care encompasses a caring approach based on humanistic values (14). In addition, researchers have pointed out the need of “employing a Life Course lens” as methodological assessment following the complex and multifactorial consequences of childhood cancer (15).

The concept of caring is essential in all healthcare, but there is no uniform definition of it (16). Still, healthcare professionals' (HCPs') actions and expectations can be studied and improved by systematically linking them to a theoretical perspective (16, 17). Swanson's Theory of Caring (1993) which is built on the foundational work of Jean Watson, exemplified an approach that promoted a practical application of caring theory. Swanson stated that “Caring is a nurturing way of relating to a valued other person, towards whom one feels a personal sense of commitment and responsibility” (18). The theory depicts caring as grounded in maintenance of a basic belief in persons, anchored by knowing the other's reality, conveyed through: knowing, being with, doing for, enabling/empowering, and maintaining belief (19). All these components are essential elements of any professional-patient relationship with the outcome in interventions that “promote, restore, and maintain optimal wellness for individuals” (20). Thus, Swanson's middle-range theory explains the link between the caring processes and the intended outcome, which is the patient's well-being (19).

Caring as perceived by people with cancer involves HCPs' having professional attitudes and skills with the intention of providing good care (21). In order to facilitate the childhood cancer survivors' everyday life during and after ending cancer-directed treatment, it is important to focus on how they experience received support. Therefore, the aim of the present study was to illustrate how childhood cancer survivors describe the role of the caring support provided by HCPs at diagnosis and during the cancer trajectory. The research group hypothesized that the quality of care delivered by HCPs during the childhood cancer trajectory has long-term psycho-social consequences. The following research questions were formulated: What aspects of caring are reported among childhood cancer survivors? Do caring experiences from childhood cancer treatment have percussions in adult life? Can Swanson's caring theory be verified among childhood cancer survivors?

## Methods and materials

### Design

An exploratory sequential mixed-method design with integrated quantitative and qualitative data was used (22), as well as descriptive and comparative analysis. A study-specific questionnaire was developed based on a qualitative approach with interviews (22) with former childhood cancer patients and a literature review (23). The present retrospective cross-sectional study collected data through this study-specific questionnaire. In the operationalization process, the results from the questionnaire were deductively analyzed with content analysis (24) using Swanson's Theory of Caring (1991; 1993) (18, 19) and the domains were statistically described and compared.

### Participants and context

#### Inclusion criteria

(1) Former patients diagnosed with solid tumors and lymphoma from 1983 to 2003, being eight to 17 years old at diagnosis, being more than 17 years old when giving their informed consent to participate, and at least three years had passed since the end of the cancer-directed treatment;
(2) Treatment modalities which included cytotoxic treatment and/or surgery and/or extra-cranial irradiation.

#### Exclusion criteria

Patients who may have received cranial irradiation.

All patients were diagnosed at the Child Cancer Centre at Queen Silvia Children's Hospital, Gothenburg, Sweden. The center has a catchment area comprising the whole of western Sweden, with a child population of about 400,000 and an annual incidence of 80–100 cases, in comparison with the annual incidence in Sweden of 300–350 cases (7). Since the beginning of 1980, the center has established a well-thought-out philosophy aiming at open communication about the disease and the adverse side effects of the treatment, and underlining the importance of comprehensive support to the patients and their families throughout the entire treatment period.

In all, 96 former patients were treated according to the above mentioned inclusion criteria and were eligible. Thus, they constituted the target group. These participants had been diagnosed with lymphoma, i.e., Hodgkin's disease (HL) and Non-Hodgkin's disease (NHL) and the rest of the group were defined as “other diagnosis”. The latter group included former patients with miscellaneous sarcomas and rare malignant disorders, for example ovarian tumors, thyroid and nasal-pharyngeal cancers. The exclusion criteria included patients who may have received cranial irradiation, i.e., primarily patients with central nervous system tumors and patients treated for leukemia. This stance was based on previous research regarding the risk of neurocognitive complications and possible difficulties in participating in a retrospective study (25).

### Data collection

The target group, i.e., a total population of 96 individuals, was invited by mail or telephone to take part in the study. When the informed consent process was ended, a study-specific questionnaire was distributed by mail and returned in pre-paid envelopes. In all, 15 eligible individuals could not be reached. Ten former patients refused participation and nine did not return the questionnaire despite being reminded three times. Overall, the individuals who did not participate represent all of the subgroups which are presented in Table 1. Thus, the final study population was considered to mirror the target group.

**Table 1:** Characteristics of the study population (*n* = 62).

Characteristics	Subgroups	Total *n* (%)
Gender	Male	28 (45.2)
	Female	34 (45.2)
Age at diagnosis	8–12 years	24 (38.7)
	13–17 years	38 (61.3)
Diagnosis	Hodgkin's disease	28 (45.2)
	Non-Hodgkin's disease	14 (22.6)
	Other solid tumours	20 (32.3)
Treatment	With extra cranial irradiation	37 (59.7)
	Without extra-cranial irradiation	25 (40.3)
Time since treatment	≤15 years	33 (53.2)
	>15 years	29 (46.8)
Age at time of study	<30 years	34 (54.8)
	≥30 years	28 (45.2)
Marital status	Married/has a partner	34 (54.8)
	Single/Not stated	24 (38.7)/4 (6.5)
Education	High school	27 (43.5)
	University	35 (56.5)
Education and healthcare	Within healthcare	24 (38.7)
	Not within healthcare	38 (61.3)
Year of diagnosis	1983–1989	21 (33.9)
	1990–2003	41 (66.1)

The questionnaire used for this study was designed specifically for the target population (26). In total, the questionnaire included 225 items with two main themes: *Quality of life in childhood cancer survivors* (*n* = 133 items) and *Caring aspects during the cancer trajectory* (*n* = 92). The answer options were presented on five-point Likert scales ranging from 1 (“I disagree”) to 5 (“I fully agree”). The content validity was determined by a process which included interviews with childhood cancer survivors and a literature review. The relevance of the items posed and the interpretability of the questionnaire were discussed with the interview contributors and experts within the field of pediatric oncology, and in relation to the concepts of quality of life (QoL) and caring aspects. An item selection and reduction process was performed using a factor analysis with the aim of strengthening the construct validity of the questionnaire. To some of the items open-ended questions were posed. The main theme *Caring aspects during the cancer trajectory* included questions such as: What memories do you have of the conversation when you were told about your disease diagnosis? Was there anything in the conversation you were not satisfied with? Which procedures were frightening or painful? A detailed description of the development of the questionnaire has been presented in a publication regarding the main theme *Quality of life in childhood cancer survivors* (23).

### Data analysis

#### Deductive analysis

**Figure 1:**
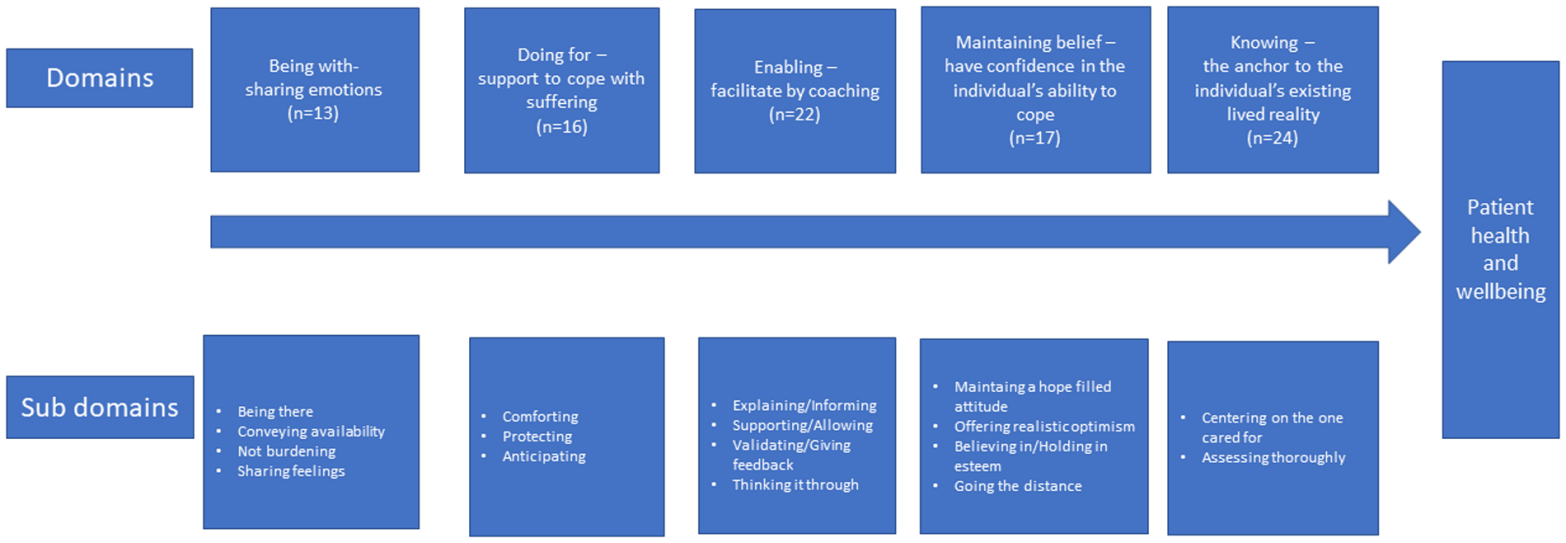
The role of caring support. Summary of the study categorization matric based on Swanson’s Theory of Caring.

Data were analyzed using qualitative content analysis with a deductive approach according to Elo and Kyngas (2008) (24). Deductive content analysis is used when the structure of analysis is operationalized on the basis of previous knowledge (24). All items in the study-specific questionnaire dealing with caring (*n* = 92 items) were read through several times to get an overview of the content. This action was performed independently by all the authors. The first step in the deductive approach was to develop a categorization matrix based on the domains in Swanson's Theory of Caring. The data were coded according to Swanson's five caring domains (knowing, being with, doing for, enabling, and maintaining belief) and appropriate associated subdomains for each caring domain. The intended outcome is the client's well-fare. The coding process in the present study was intended to develop and use related concepts for each domain, to illustrate how the survivors described the caring support provided by HCPs during the period of illness. The posed related concepts are presented in Figure 1 with the number (n) of items within each domain. In Elo and Kyngas (2008) content analysis model, a domain is described as Generic category and a sub domain as Sub category (24).

### Statistics

The descriptive statistics consist of frequencies, median values, mean values and standard deviations. Before the analyzes were performed, all negative items were reversed. Chi-square statistics were obtained in order to compare proportions of categorical variables between demographic data/characteristics. Fisher's exact test was used when more than 15% of the cells had an expected value of <5. To compare values between two independent variables, the Mann-Whitney test was used, while the Kruskal-Wallis test was used to compare three independent variables. Statistical significance was obtained at the level of *p* < 0.05.

The coded data identified by the previously described deductive content analysis (24) was analyzed using principal component analysis. Kaiser-Meyer-Olkin (KMO) and Bartlett's test of sphericity were performed. The obtained value for KMO was 0.624 and for Bartlett's test 0.000, which indicates a matrix capable of factorization. The extraction method was based on five fixed factors. Items (descriptors) with correlations above 0.5 were organized into factor groups describing their correlation to the five domains in Swanson's Theory of Caring (19). Reliability within each domain (factor) was verified by calculating the internal consistency, using the Cronbach's alpha value, for each domain separately (26). A criterion of 0.60–0.90 is proposed to be a good internal consistency, in exploratory studies (27). The statistical analyzes were performed using IBM SPSS Statistics version 22.0 (IBM Corp, Armonk, New York, USA).

### Ethical considerations

The participants were given oral and written information about the study before they gave their informed consent to participate. The Regional Research Ethics Board, Gothenburg Sweden, approved the study (Dnr 289-07).

## Results

### Study participants

The final study group consisted of 62 persons (34 women and 28 men). The mean age at diagnosis was 12.9 years (range 8–17, SD 2.3, median value 13 years). The mean current age of the participants was 28.7 years (range 18–45, SD 6.3, median value 28.5 years) and the mean time passed since treatment was 15.7 years (range 4–28, SD 2.4, median value 15 years). The response rate was 65%. Sub-group analyzes were performed including gender, being a school-age child (8–12 years) or adolescent (13–17 years) at the time of diagnosis, diagnosis (HL, NHL, other solid tumors), and treatment with or without extra-cranial irradiation. Furthermore, sub-group analyzes regarding time since treatment (cut off: median value 15 years), being a child or a young adult, i.e., younger than 30 years (28), or older than 30 years when participating in the study, living with/without a partner, education at university level or not and having an education within healthcare or not. The year of diagnosis was defined in two categories, 1983–1989 or 1990–2003, i.e., before or after the early 1990s. In the beginning of the 1990s there was a shift in treatment modality vis-à-vis patients with HL, in particular with the main goal of creating protocols with reduced radiotherapy (29) (Table 1).

### Categorization matrix based on Swanson's middle-range theory of caring

A summary of the study categorization matric based on Swanson's Theory of Caring, with the distribution of the total number of descriptors from each domain (study-specific questionnaire) and the sub domains identified in this study is presented in Figure 1. The contributing descriptors with values above 0.5, for each of the five latent factors/domains together with the strength of the correlation are presented in Table 2. In total, 26 descriptors out of 92 fulfilled the correlation criteria. The total explained variance was 62.4%. Few participants answered the open-ended questions and therefore, these questions were not analyzed separately with qualitative methods. The free worded answers have partly influenced the analysis process and the interpretation of data in the discussion.

**Table 2:** Description of the domains, sub domains and descriptors with values above 0.5, based on Swanson's Theory of Caring.

Factor	Domains	Sub domains	Descriptors with values above 0.5
1	Being with-sharing emotions	Conveying availability	I got support from the nurses
		Not burdening	The staff showed respect for me
		Sharing feelings	The staff at the department were engaged
		Sharing feelings	I felt personally taken care of
		Being there	I could openly talk to the nurses
		Sharing feelings	I experienced that the doctor was sincere towards me
		Being there	I got good contact with the treating doctor
		Sharing feelings	I felt that I could totally trust the doctor
2	Doing for—support to cope with suffering	Comforting	I was very worried about the diagnosis right then
		Comforting	The first day after the conversation I felt very depressed
		Anticipating	I was afraid I could be seriously ill before I applied for a doctor
		Anticipating	I didn't care about my health, but felt healthy
		Protecting	Several investigation procedures were frightening
3	Enabling –facilitate by coaching	Supporting/Allowing	I was told what was going to be done
		Informing/Explaining	I received information from other healthcare professionals
		Validation, giving back	I know the name of the disease, as well as the facts about it
		Thinking it through	I had the opportunity for several information conversations
		Informing/Explaining	I got information from the doctor
4	Maintaining belief –have confidence in the individual's ability to cope	Offering realistic optimism	I understood the meaning of the diagnosis
		Maintaining a hope filled attitude	I had no idea what they were talking about and what it meant
		Going the distance	I understood the seriousness of the situation
		Maintaining a hope filled attitude	I understood the diagnosis only after further information calls
		Believing in/holding in esteem	After information from various professionals, I understood the diagnosis
		Going the distance	The regular post-treatment controls raised concerns
5	Knowing—the anchor to the individual's existing lived reality	Centering on the one cared for	The diagnosis call took place in a quiet environment
		Assessing thoroughly	The doctor took the time to inform about the diagnosis

### The concept of caring and sub-group analysis (characteristics of the study population)

#### Factor 1 being with—sharing emotions

Being with—sharing emotions was the most prominent loading categorical factor indicator. This domain reflects the caring process of being with. According to Swanson (Swanson, 1993; Wojnar, 2006) (19, 30) being with includes being genuinely present for others in order to convey that their experiences have significance. The items included in this domain, from the study-specific questionnaire, were linked to the subdomains; being there, conveying availability, not burdening, and sharing feelings (Table 2). The Cronbach's alpha value was equal to 0.89.

Childhood cancer survivors belonging to the older age group (>30 years) rated the role of being with—sharing emotions as more salient, in comparison with young adults (<30 years), (*p* = 0.041) (Tables 3A–C).

**Table 3A:** Domains and sub-groups characteristics in comparison (Mann-Whitney).

Domains defined in this study	Characteristics				
	Median	Q1–Q3	Median	Q1–Q3	*p*-value
	Male		Female		
Sharing emotions	5.0	4.1–5.0	5.0	4.3–5.0	.771
Support to cope with suffering	3.0	2.0–4.0	3.0	2.0–3.7	.926
Facilitate by coaching	4.3	4.0–5.0	4.0	3.9–5.0	.533
Have confidence in the individual's ability to cope	2.0	1.5–3.0	3.0	2.1–3.6	.021*
The anchor to the individual's existing lived reality	4.2	4.0–5.0	4.5	4.0–5.0	.794
	Year of diagnosis,1983–1990		Year of diagnosis, 1990–2003		
Sharing emotions	5.0	4.7–5.0	5.0	4.0–5.0	.123
Support to cope with suffering	3.0	2.2–4.0	2.5	2.0–3.6	.162
Facilitate by coaching	4.0	3.7–5.0	4.3	4.0–5.0	.377
Have confidence in the individual's ability to cope	3.0	2.1–4.0	2.5	1.9–3.5	.228
The anchor to the individual's existing lived reality	4.0	3.7–5.0	4.5	4.0–5.0	.310
	Age at diagnosis, 8–12 years		Age at diagnosis, 13–17 years		
Sharing emotions	5.0	4.1–5.0	5.0	4.0–4.5	.843
Support to cope with suffering	2.8	2.0–3.5	3.1	2.0–4.0	.211
Facilitate by coaching	4.2	4.0–5.0	4.3	4.0–5.0	.657
Have confidence in the individual's ability to cope	3.0	2.4–4.0	2.2	1.9–3.0	.048*
The anchor to the individual's existing lived reality	4.5	4.0–5.0	4.2	4.0–5.0	.275
	Treatment, extra-cranial irradiation		Treatment, no extra- cranial irradiation		
Sharing emotions	5.0	4.0–5.0	5.0	4.5–5.0	.445
Support to cope with suffering	2.5	1.8–3.5	3.1	2.2–4.0	.071
Facilitate by coaching	4.3	4.0–5.0	4.3	4.0–5.0	.831
Have confidence in the individual's ability to cope	2.2	1.5–3.0	3.0	2.6–4.0	.004*
The anchor to the individual's existing lived reality	4.5	4.0–5.0	4.2	4.0–5.0	.546
	Time since treatment ≤15 years		Time since treatment >15 years		
Sharing emotions	5.0	4.0–5.0	5.0	4.5–5.0	.183
Support to cope with suffering	2.5	1.5–3.5	3.0	2.3–4.0	.045*
Facilitate by coaching	4.3	4.0–5.0	4.3	4.0–5.0	.759
Have confidence in the individual's ability to cope	2.7	1.5–3.5	2.9	2.0–3.2	.594
The anchor to the individual's existing lived reality	4.5	4.0–5.0	4.2	3.8–5.0	.158

* Significant differences.

**Table 3B:** Domains and sub-groups characteristics in comparison (Mann-Whitney).

Domains defined in this study	Characteristics				
	Median	Q1-Q3	Median	Q1-Q3	*p*-value
	Age at time of study, <30 years		Age at time of study, ≥30 years		
Sharing emotions	4.8	4.0–5.0	5.0	4.7–5.0	.041*
Support to cope with suffering	2.4	1.5–3.5	3.1	2.5–4.0	.010*
Facilitate by coaching	4.7	4.0–5.0	4.0	3.8–5.0	.182
Have confidence in the individual's ability to cope	2.6	1.5–3.5	2.8	2.0–3.9	.446
The anchor to the individual's existing lived reality	4.5	4.2–5.0	4.0	3.6–4.8	.013*
	Married/has a partner		Single/not stated		
Sharing emotions	5.0	4.0–5.0	5.0	4.5–5.0	.405
Support to cope with suffering	3.0	2.0–3.8	2.8	1.6–4.0	.728
Facilitate by coaching	4.0	3.8–5.0	5.0	4.0–5.0	.010*
Have confidence in the individual's ability to cope	3.0	2.1–3.5	2.0	1.1–3.0	.041*
The anchor to the individual's existing lived reality	4.5	4.0–5.0	4.2	4.0–5.0	.766
	Education, High school		Education, University		
Sharing emotions	5.0	4.0–5.0	5.0	4.5–5.0	.426
Support to cope with suffering	3.2	2.0–4.0	3.0	2.0–3.5	.339
Facilitate by coaching	4.3	4.0–5.0	4.3	4.0–5.0	.804
Have confidence in the individual's ability to cope	3.0	2.0–4.0	2.4	2.0–3.0	.061
The anchor to the individual's existing lived reality	4.5	4.0–5.0	4.2	4.0–5.0	.378
	Education, within healthcare		Education, not within healthcare		
Sharing emotions	4.8	4.0–5.0	5.0	4.5–5.0	.116
Support to cope with suffering	3.1	2.0–4.0	2.8	1.9–3.5	.266
Facilitate by coaching	4.2	3.8–5.0	4.3	4.0–5.0	.759
Have confidence in the individual's ability to cope	2.9	1.6–3.5	2.8	2.0–3.5	.994
The anchor to the individual's existing lived reality	4.5	4.0–5.0	4.2	4.0–5.0	.306

* Significant differences.

**Table 3C:** Domains and sub-group characteristics in comparison (Kruskal-Wallis).

Domains defined in this study	Characteristics						
	Median	Q1–Q3	Median	Q1–Q3	Median	Q1–Q3	*p*-value
	Hodgkin lymphoma		Non-Hodgkin lymphoma		Other solid tumors		
Sharing emotions	5.0	4.0–5.0	5.0	4.5–5.0	5.0	4.4–5.0	.753
Support to cope with suffering	2.8	2.0–4.0	3.1	2.0–4.0	2.8	2.0–3.6	.754
Facilitate by coaching	4.6	4.0–5.0	4.0	4.0–4.5	4.6	3.8–5.0	.619
Have confidence in the individual's ability to cope	2.0	1.5–3.0	3.0	2.4–4.0	2.9	2.2–3.9	.134
The anchor to the individual's existing lived reality	4.5	4.0–5.0	4.2	4.1–5.0	4.1	4.1–5.0	.638

#### Factor 2 doing for—support to cope with suffering

Doing for—support to cope with suffering was the second most prominent loading factor. This factor reflects Swanson's process of doing for (19, 30) which consists of doing for other persons what they would do for themselves, if possible. Items in this study cover the subdomains of comforting, protecting and anticipating (Table 2). The Cronbach's alpha value was equal to 0.79.

Survivors with long-term follow-up, defined as more than 15 years post-treatment, and those in the older age group (more than 30 years) when participating in the study, reported higher rating regarding HCPs doing for others what they would do for themselves, compared to those with a shorter time of follow-up and who were young adults (*p* = 0.045 and *p* = 0.010, respectively) (Tables 3A–C).

#### Factor 3 enabling—facilitate by coaching

Enabling—facilitate by coaching corresponds to Swanson's concept of enabling (19, 30) and entails assisting individuals to acquire the tools they need to be able to care for themselves, including periods with life-transitioning events. The study-specific items in this domain are related to the subdomains of explaining/informing, supporting/allowing, validating/giving feedback, and thinking it through (Table 2). The Cronbach's alpha value was equal to 0.77.

This caring process was rated higher among individuals living with a partner compared to those who were single (*p* = 0.013) (Tables 3A–C).

#### Factor 4 maintaining belief—have confidence in the individual's ability to cope

Having confidence in an individual's ability to cope is in line with Swanson's concept of maintaining belief (19, 30), which incorporates a fundamental belief in people and their ability to make it through events and transitions and to face a future with purpose. The subdomains of maintain a hopeful attitude, offering realistic optimism, believing in/holding in esteem, and going the distance (Table 2) are present in this study. The Cronbach's alpha value was equal to 0.70.

Participants who lived with a partner conveyed higher rating about having been assisted with tools to take care of themselves and the ability to cope with difficulties, in comparison with persons who were single (*p* = 0.041). An increased vulnerability regarding the ability to cope was more salient among men compared to women (*p* = 0.021), among those who were treated during adolescence (13–17 years) related to schoolchildren (8–12 years) (*p* = 0.048) and among participants who had been treated with extra-cranial irradiation, in comparison with no extra-cranial irradiation (*p* = 0.004) (Tables 3A–C).

#### Factor 5 knowing—the anchor to the individual's existing lived reality

The anchor between the HCPs' and the individual's existing lived reality is associated with the caring process of knowing (19, 30). Knowing can be defined as the starting point for the striving “to understand an event as it has meaning in the life of another person” (30). The items included reflect the subdomains of centering on the one cared for and assessing thoroughly (Table 2). The Cronbach's alpha value was equal to 0.89.

The HCPs' willingness to understand the sick child's situation was highlighted particularly in the older age group compared to those being less than 30 years of age when they participated in the study (*p* = 0.013) (Tables 3A–C).

No significant differences were found within the following sub-groups; year of diagnosis (Table 3A), education (high school/university) (Table 3B), education within or not within healthcare (Table 3B) and the cancer diagnosis in childhood (Table 3C).

## Discussion

The results of this study highlight that through the lens of Swanson's Theory of Caring, childhood cancer survivors experience the HCPs as being emotionally present, carrying out actions, and involving children in their treatment and follow-up care.

The caring process of being with, in the present study defined as “Being with—sharing emotions”, had the most prominent loading and showed how the survivors appreciated care, engagement, openness and trust in HCPs. Being with can be considered as a way of deepening the role of knowing, i.e., by trying to see events from the perspective of the person affected. In the present study the older age group of survivors rated the role of HCPs being emotionally present, as well the HCPs' willingness to understand the sick child's situation, as more salient in comparison with young adults. The long-term survivors' lives involve a process of struggle to overcome numerous difficulties. According to previous studies, cancer survivors need pronounced support and information to get life back to normal after treatment (31–33). For participants belonging to the older age group, several years had passed since they were diagnosed with childhood cancer. These participants had high ratings regarding the role of HCPs being emotionally present to comfort worried and depressed children/adolescents at the time of diagnosis, as well as giving support to cope with frightening procedures. These actions probably reflect the experiences of the role of creating good relationships and HCPs being available during illness and after treatment. This is in line with the study of Cantrell and Matula (2009) (34), which points to the fact that it is not exclusively the extraordinary efforts that leave a mark; rather, simple acts of caring are meaningful. Such actions can mediate comfort during treatment, facilitate participation in everyday life, and also have implications in a long-term perspective. In addition, when patients feel they are seen and heard by the HCPs they are likely to more actively participate in their own care and if needed seek care (14).

In the present study, vulnerability was related to gender, age at diagnosis, marital status and oncological treatment in childhood, regarding the individual's ability to cope with the impediments. These data confirm previously identified risk groups for susceptibility, namely persons living alone, being male, falling ill as a teenager, and having been treated with radiotherapy (35, 36). Previous research has shown that survivors of childhood cancer live with worries and uncertainty (37), but also rate high on positive life changes and sense of purpose (3). Furthermore, they may have grown positively due to their negative past experience (3). The role of healthcare providers is crucial as the patient's susceptibility can increase or decrease related to the manner in which HCPs interact with the patient (38). Therefore, it is vital to address the fact that patients’ and professionals' perceptions of the overall caring can differ significantly, as well as perceptions of individual behaviors (16). Thus, it is fundamental that children and adolescents are seen, heard and listened to in order to have a healthy life. The meeting with the HCPs will be important and will reflect how the children/adolescents experience their ongoing cancer trajectory and their opportunities to dare to look ahead. Consequently, it can be considered important that the HCPs train in caring, based on an approach with a comprehensive theory to be able to define and measure verbal and non-verbal caring and non-caring behaviors. The Caring Behavior Coding Scheme (the CBCS), based on Swanson's Theory of Caring, has been proposed to fulfil such a purpose and could be a way to assess care efforts (39).

### Involving the child

Involving the child is a process which starts at the time of diagnosis, runs through the entire treatment period, the transition to a healthy life, and goes beyond post-treatment, with long-term implications. Research has shown that the desire to participate and to be involved applies to all children, even the very smallest treated for malignant diseases (40).

The study results highlight the HCPs' efforts to try to go the whole distance by understanding, sharing emotions, performing actions to help the child to cope with suffering, and sustaining faith in the child's capacity. Focusing on the child's participation and the consequences in a long-term perspective is crucial. Previous studies within pediatric oncology caring science have well-being and QoL as central themes, but mainly from the view of the parents and rarely show the child's or the survivor's perspective (41, 42).

Childhood cancer survivors require age-appropriate and flexible care. They experience social withdrawal and awkwardness due to adaptation difficulties caused by social life interruption, family issues, social prejudice, and discrimination. In our previous publication with the theme of QoL in childhood cancer survivors, the results revealed the importance of social support for well-being during the child's active treatment phase (23). The physical and social struggles may result in experiences of mental stress and psychological withdrawal followed by rebound (37). It is important to watch for these signs and to provide early support to survivors so that their daily life and development are not hindered by the treatment or its side effects, and to offer long-term support focusing on individual patient characteristics (43). In the long-term perspective, the need for psychological support is highlighted (23). Regarding psychological side effects, there is a call for support in the transition back to normal life after the cancer experience (44). In addition, Barnett et al. (2016) found that young cancer survivors' experiences are nuanced, with interacting variables contributing to post-treatment outcomes (44). In line with the model of participation described by Imms et al. 2017, the two concepts of attendance in activities as well as involvement in activities are essential (45). Therefore, providing good care and being caring are central factors when highlighting the role of participation. In the context presented in this study these actions can be defined as sharing the child's emotions and experiences, being with the child, doing things for the child that he/she cannot do for himself/herself, and trying to make life easier for the child by informing and explaining. It is also important to sustain faith in the child and the child's ability to cope with the circumstances and the future, as well as wanting to find out the child's lived reality. All these actions represent a way of reflecting the willingness of the HCPs to facilitate life for each child. When the HCPs enable the child to be present in his/her own treatment, through severe conditions, and support the child in everyday life, then the HCPs will help the child to be involved in his/her own life and promote the child's personal health.

### Implications for healthcare professionals

Swanson's Theory of Caring includes processes which at a deeper level suggest actionable interventions that make the theory-practice construction understandable and suitable for clinicians (46). HCPs in pediatric oncology care need to be aware of the effects of the disease on the whole family, i.e., the role of systems for care improvement, provision of QoL, education and support, and empowerment of children and families (41). Above all it is important to underline that the role of participation goes beyond treatment and childhood. Therefore, understanding and awareness of late effects and how survivorship is applied in the context of childhood cancer survivors is critically important to the practice of HCPs. This insight will offer them new possibilities for providing guidance, support, and assistance in enhancing outcomes for childhood cancer survivors and their families (47). In practice, this means that professionals need to help survivors in the transition to a new normality, create hope by nurturing a trustful relationship, support coping by giving knowledge and information, decrease distress, anxiety and pain, show genuine interest in the persons' lives outside the hospital, and have a plan for follow-up (48). The practical transfer of knowledge can advantageously take place *via* information brochures and educational material with a person-centered adaptation. To improve HCPs' caring behavior, with the aim of increasing the patient's well-being, the above mentioned coding scheme (the CBCS) could be useful (39). However, all efforts presuppose that children are actively involved and listened to, i.e., they are partners in shared decision-making from the point of diagnosis (49).

### Strengths and limitations

The study has been performed with a cohort of former patients treated at the same childhood cancer center, which has had a clear care philosophy over decades. Still, the study population is heterogeneous and in retrospective studies researchers always need to take into account the role of time and how the study participants' memory can influence the study results. Based on the demographic data (Table 1) we can conclude that the invited participants who did not answer represent all of the defined subgroups. However, we do not know how these individuals would have answered the questionnaire. We need to be aware of the risk of the possible systematic error and conclusions need to be drawn with caution. To avoid the risk of recall bias another approach is to perform longitudinal studies, quantitative as well as qualitative. Preferably, the study starts at diagnosis and with focus on the role of participation in everyday life during the child's cancer treatment trajectory and at follow-up.

## Conclusion

With a large number of children surviving childhood cancer worldwide, numerous investigations have assessed psychological and social adjustments among childhood cancer survivors. The results from this study underline the role of caring from a long-term perspective. The results yield improved understanding of the population of childhood cancer survivors, help draw attention to their challenges, and contribute to their more effective integration into society, helping those individuals' live healthy and positive lives (3, 37). The journey to this healthy life starts by promptly involving the sick child. The role of participation persists through and beyond treatment with a lifelong perspective to foster autonomy for long-term survivorship.

## Data Availability

The data sets presented in this study include personally identifying information that present risks to confidentiality. According to the ethics approval the data cannot be shared and therefore, we do not include an email address where requests for the data set can be sent.
